# Ipsilateral Radial Head Dislocation And Proximal One-Third Radial Shaft Fracture In An Adult: A Case Report

**DOI:** 10.2174/1874325001812010189

**Published:** 2018-05-31

**Authors:** Jagdeep Singh, Anoop Kalia, Anshul Dahuja

**Affiliations:** 1 Department of orthopaedics, Guru Gobind Singh Medical College and Hospital, Faridkot, Punjab, India; 2 Department of Orthopaedics, Guru Nanak Dev Super Speciality Hospital, Tarn taran, Punjab, India

**Keywords:** Isolated, Radial head, Dislocation, Ipsilateral, Fracture shaft Radius, Proximal

## Abstract

**Introduction:**

Dislocation of the radial head in adults is quite uncommon. A simultaneous dislocation of the radial head with a fracture of ipsilateral shaft radius without any other associated injury is even rare.

**Case Presentation:**

We are reporting a case of a young adult male who was operated for proximal one-third radial shaft fracture at some peripheral centre by Open Reduction and Internal Fixation (ORIF), but came to our centre on the fourth post-operative day with complaints of painful restricted movements of the elbow joint. On careful look at the postoperative x-ray, radial head was found to be dislocated. Radial head dislocation was reduced under general anesthesia and at 2 years follow up, patient fracture has fully united having good functional outcome.

**Conclusion:**

Traumatic dislocation of radial head with ipsilateral fracture shaft radius is a rare injury in adults and it is very important to timely diagnose it and manage it appropriately in order to give good functional outcome to the patient.

## 1 INTRODUCTION

Radial head dislocations are usually associated with either complete elbow dislocation or fracture of proximal one-third ulnar shaft as in monteggia complex [1, 2]. Isolated post-traumatic radial head dislocations with no other associated injuries are common in children and usually labeled as monteggia lesion [3] but rare in adults. If neglected, this injury can have many untoward complications like the restriction of forearm supination and pronation, secondary degenerative arthritis of elbow and distal radioulnar joints [3]. Dislocation of radial head with ipsilateral radial shaft fracture is even rarer; to the best of our knowledge very few cases have been reported in the literature [4-6].

## 2 CASE PRESENTATION

**Fig. (1):**
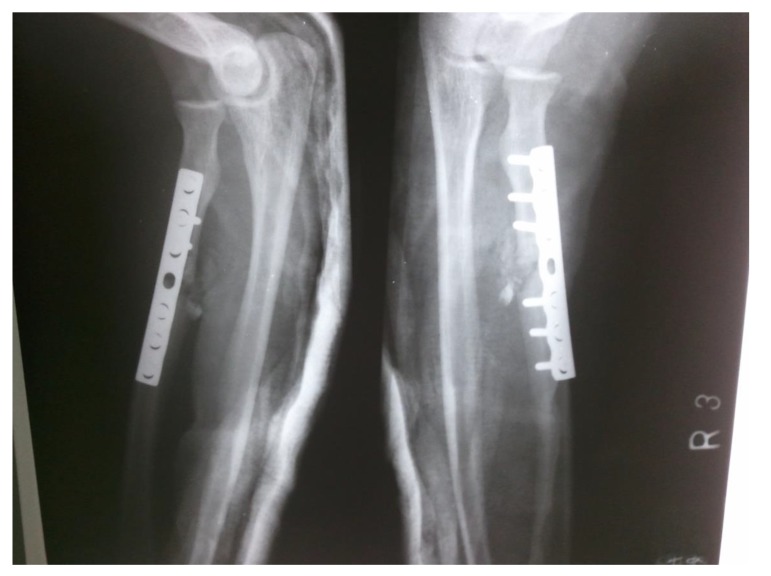
ORIF of proximal 1/3^rd^ radial shaft fracture done using 7 hole DCP with radial head lying dislocated anterolaterally.

**Fig. (2):**
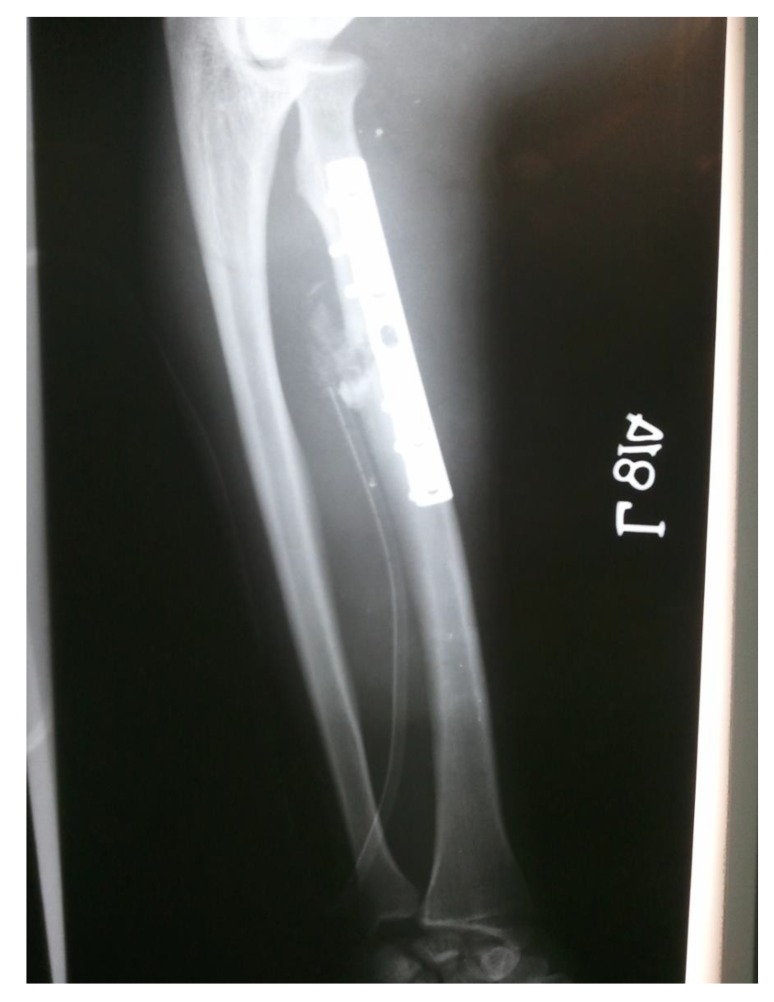
Showing intact distal radio ulnar joint.

**Fig. (3):**
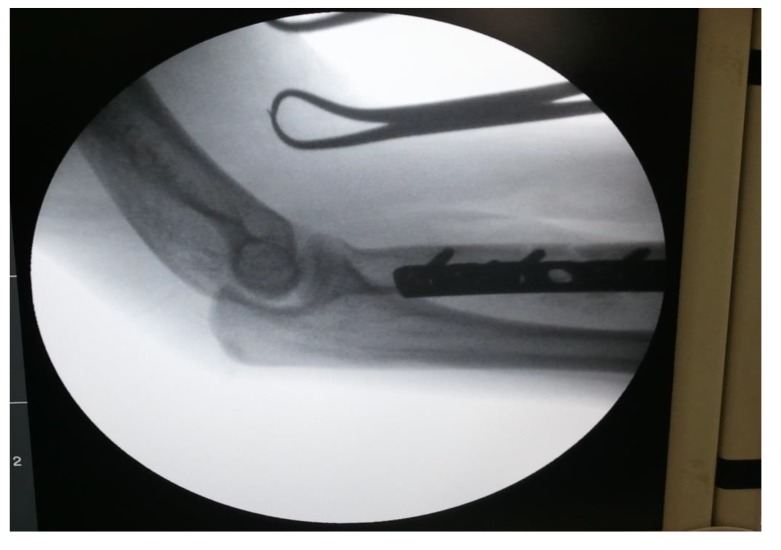
C-arm picture showing dislocated radial head after being reduced back to its normal position in the lateral view.

**Fig. (4):**
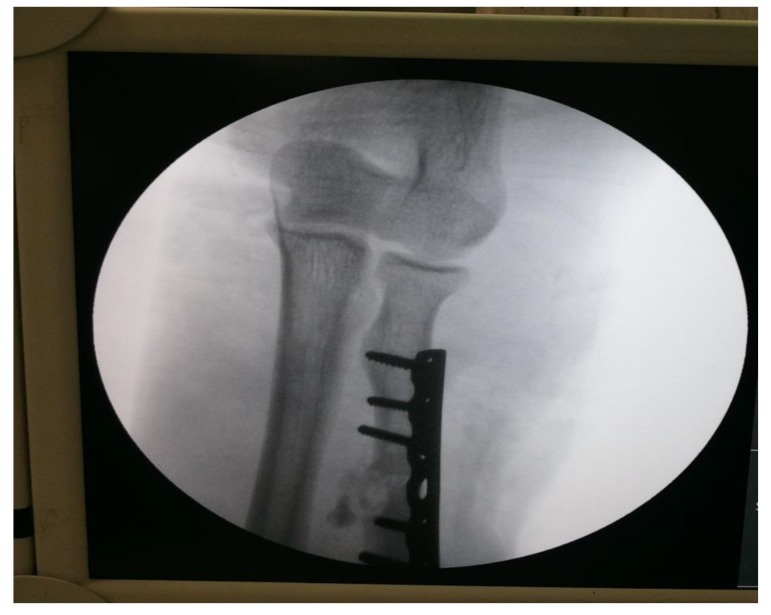
C-arm picture showing dislocated radial head after being reduced back to its normal position in the anteroposterior view.

**Fig. (5):**
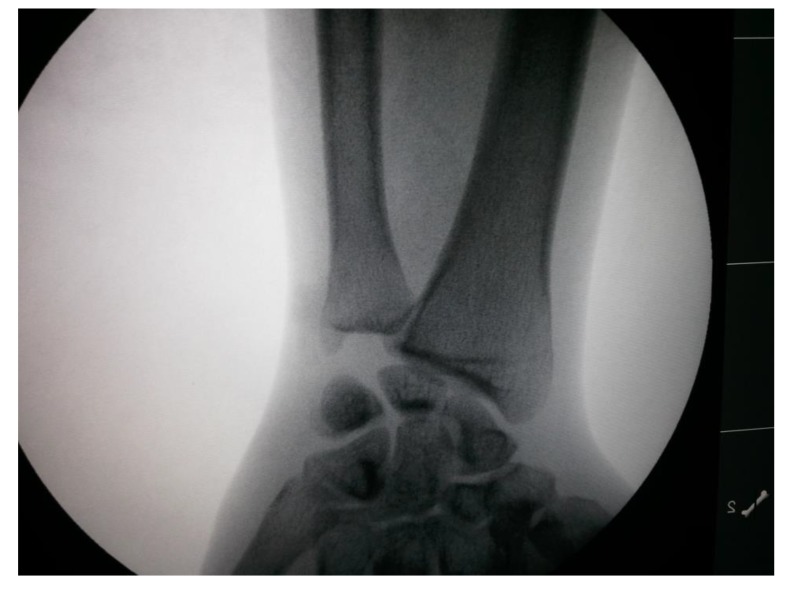
Showing fluoroscopic image of intact distal radioulnar joint.

**Fig. (6):**
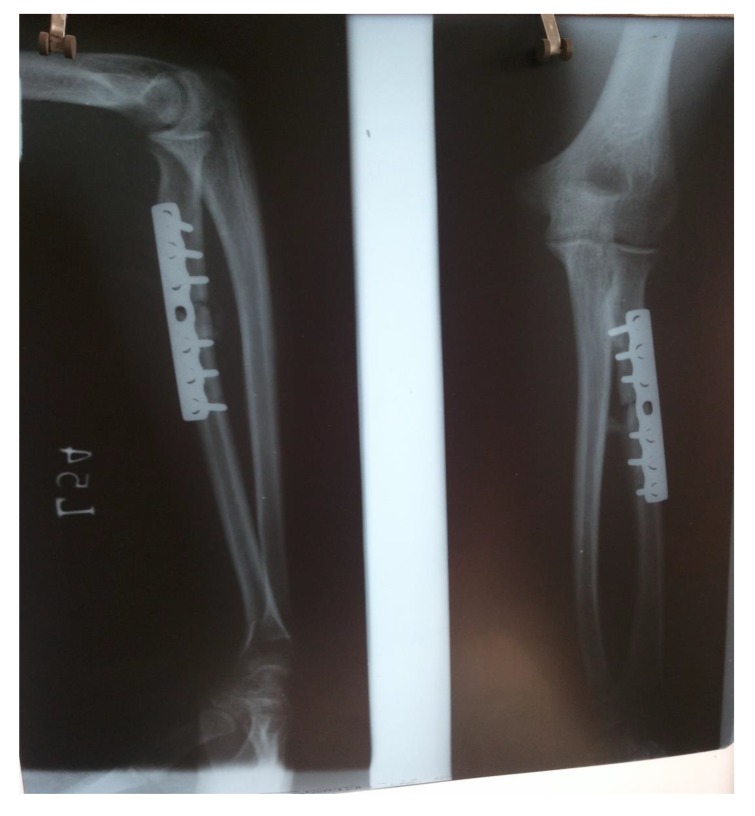
At 6wks follow up-showed good reduction of radial head.

**Fig. (7):**
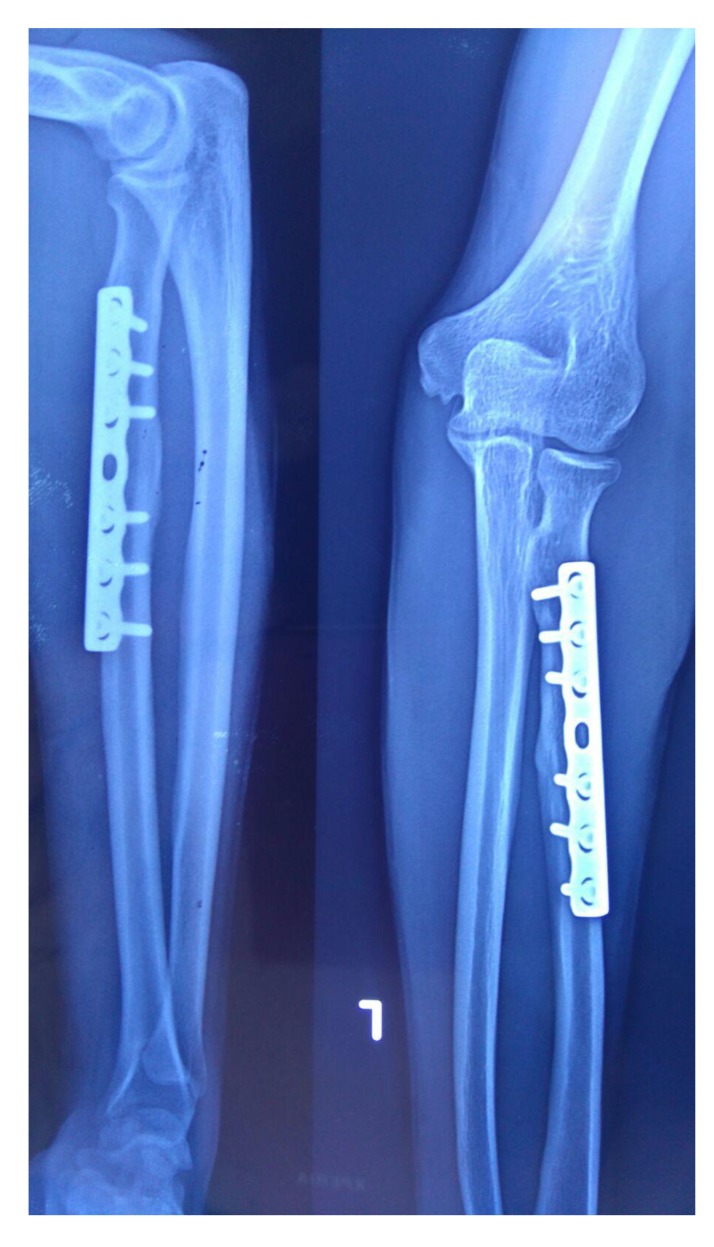
Solid union at the fracture site at the end of two years.

A young adult male presented to Out Patient department of our hospital as follow up of surgery for isolated radius shaft fracture that was done 4 days ago at some other private institution. He was complaining of painful and restricted movements at the elbow joint after four days of surgery. The patient reported that he had fallen from stairs and sustained injury to the forearm. There was tenderness and swelling over proximal forearm region. Movements at elbow joint were painful and restricted. Patient went for opinion elsewhere and he was diagnosed as a case of fracture

proximal one-third radius shaft with no neurological deficit. Patient was taken up for surgery on the same day and Open Reduction and Internal Fixation (ORIF) with 7 holed small DCP (dynamic compression plate) were performed. He was also told by operating surgeon to start mobilization of forearm from the first postoperative day but he was unable to do it. The patient did not have any preoperative x-rays record. His post-op x rays showed fixation of fracture proximal to one third radius using a 7 holed DCP. On careful observation it was seen that radial head was lying dislocated anterolaterally (Figs. **1**, **2**) with intact distal radioulnar joint. The patient was counselled about the future consequences if the radial head was left unreduced. He was also counselled about revision surgery. After proper explanation and with informed consent, the patient was taken up in operation theatre. Keeping a requirement of revision surgery in mind, after a proper consent, the patient was taken under general anaesthesia. After proper painting and draping, closed manipulation to reduce the radial head was tried. Forearm was supinated and pressure was applied medially and posteriorly over the anterolateral aspect of proximal radius along with pulling of forearm and when checked under fluoroscopy in both anteroposterior and lateral views, it was seen that the dislocated radial head had reduced back to its normal position (Figs. **3**, **4**). Distal radioulnar joint was also checked under fluoroscopy and found to be stable (Fig. **5**). Elbow was immobilized with POP (Plaster of Paris) splint in supination and 90-degree flexion for 3 weeks. POP back slab was removed after 3 weeks and check x-rays were done which showed reduced radial head. Active movements except pronation and supination were started under the supervision of a physiotherapist. At 6 weeks follow up, active supination and pronation movements were started and x-ray showed reduced radial head (Fig. **6**). The patient was followed up to 2 years at 3 monthly intervals and at final 2 years follow up, there was no pain over elbow as solid union of fracture was there with radial head in the reduced position (Fig. **7**).

## 3 DISCUSSION

Radial head dislocations are generally associated with fractures of proximal ulna. This is known as monteggia fracture dislocation. Such injuries are quite common in children [1, 2]. Isolated radial head dislocations without associated ulna fracture or elbow dislocations in adults is a rare injury. An extensive search of the literature revealed only a few cases reported by various authors [7-10].

However, radial head dislocation with associated radial shaft fracture is even rarer to the best of our knowledge. Very few cases have been reported in the literature. Mehra **et al**. [4] in their case report mentioned about a young adult male who was 25 years old and sustained anteroinferior dislocation of radial head with associated radial shaft fracture. The mode of injury, in that case, was assault by a wooden stick. A similar case of traumatic radial head dislocation with associated radial shaft fracture was reported by Cherif **et al**. [5]. Simpson **et al**. [6] reported a case in which the patient had anterior dislocation of radial head with associated fracture of ipsilateral shaft radius, the mechanism of injury was excessive pronation of forearm. In children also similar case has been reported in which there is ipsilateral radial shaft fracture with radial head dislocation [11]. A unique case describing the fracture of distal end radius with dislocation of ipsilateral radial head has been reported by Vinay *et al*. [12]. In that case, closed reduction was performed for both injuries and elbow was immobilized in 90 degree flexion and supination pop slab was removed after three weeks and movements were started. At 2 years follow up, the patient was pain-free and no instability was present at elbow joint. Shamian B [13] described an uncommon case of an isolated radial shaft fracture with an unreducable posterior dislocation of the radial head and associated rupture of the lateral collateral ligament of the elbow.

Various mechanisms have been described for the radial head dislocations. The most common mechanism is falling on an outstretched hand fully extended or semiflexed along with varus stress applied at the elbow. The most important factor in maintaining the stability of radial head is annular ligament. The proximal radio-ulnar joint is most stable in supination. In supination, the interosseous membrane, annular ligament and anterior fibers of quadrate ligament are taut and the contact between proximal radius and ulna is maximal. The cadaveric studies have shown that anterior dislocations of radial head occur with forearm in extreme supination by completely severing anterior capsule and annular ligament and applying anteriorly-directed force to the posterior aspect of radial head [14]. The laterally dislocated radial head often reduces by just supinating the forearm [14] and also depending on whether radial head is dislocated anteriorly or posteriorly, the force can be applied in that direction to reduce it. There is a difference in the opinion amongst various authors as to immobilize the radial head in supination or pronation after reduction. Majority of authors believe in immobilizing radial head in flexion and supination [14, 15]. While some authors believed in immobilizing in flexion and pronation [7, 8, 16].

We believe that in our case, the mechanism of injury was fall from the height on the outstretched hand with forearm semi-flexed and in extremes of supination along with varus stress at elbow joint. This leads to the rupture of annular ligament causing the radial head to dislocate antero laterally along with fracture shaft radius on the same side. Since the described injury is rare, there are no established guidelines as to how to manage it. The main aim should be a concentric reduction of radial head. Open reduction of radial head should be done if there is interposed fibers of annular ligament or anterior capsule.

## CONCLUSION

This case report highlights the importance of keen observation, detailed clinical and radiological examination of both proximal and distal joints. Thus it can be summarized that traumatic dislocation of radial head with ipsilateral fracture shaft radius is a rare injury in adults and it is very important to timely diagnose it and manage it appropriately in order to prevent disability which is inevitable if it is missed.
